# Sex-specific comparison of clinical characteristics and prognosis in Crohn’s disease: A retrospective cohort study of 611 patients in China

**DOI:** 10.3389/fphys.2022.972038

**Published:** 2022-09-29

**Authors:** Zhaoshi Liu, Xiaoyin Bai, Huimin Zhang, Zheng Wang, Hong Yang, Jiaming Qian

**Affiliations:** Department of Gastroenterology, Peking Union Medical College Hospital, Peking Union Medical College, Chinese Academy of Medical Sciences, Beijing, China

**Keywords:** Crohn’s disease, sex, disease behaviour, surgery, survival analysis

## Abstract

**Background:** Real-world data on the impact of sex on the disease progression and prognosis of Crohn’s disease (CD) from large-scale Chinese cohorts are lacking.

**Aims:** This study aimed to evaluate sex disparities in the clinical characteristics of, disease progression behaviours of and surgery-related risk factors for CD.

**Methods:** A retrospective cohort study comprising 611 patients consecutively diagnosed with CD at Peking Union Medical College Hospital from January 2000 to December 2020 was conducted. Multivariate Cox regression and survival analyses was performed to assess the risk factors for disease progression and CD-related surgery in sex subgroups.

**Results:** Male sex was an independent protective factor against multisystemic extraintestinal manifestations [EIMs] (HR: 0.52, *p* = 0.03) and a risk factor for intestinal perforation (HR: 1.85, *p* = 0.01). Male patients had longer EIM-free survival (*p* = 0.024) and shorter intestinal perforation-free survival (PFS) than females (*p* = 0.012). Of the 397 patients with the A2 classification, male patients had a higher risk of CD-related surgery (HR: 1.80, *p* = 0.028) and shorter surgery-free survival (SFS) than female patients (*p* = 0.04).

**Conclusion:** Sex disparities in disease progression and outcomes of CD were revealed in a single Chinese centre. Male sex was independently associated with worse disease progression and prognosis including multisystemic EIMs and perforation, which suggests the need for individualized management according to risk classification.

## 1 Introduction

Crohn’s disease (CD), a type of inflammatory bowel disease (IBD), is recognized as a chronic inflammatory disorder of the gastrointestinal tract with specific stenotic or penetrating disease behaviour. The pathogenesis of CD is complex, and genetic susceptibility, innate and adaptive immune system dysregulation, environmental factors, and intestinal ecological disorder are involved (Shah et al., 2018). In some chronic immune-mediated diseases, the incidence and prevalence are discordant between female and male patients, indicating a potential biological role of sex hormones based on sex-related genes in disease pathogenesis (Fairweather et al., 2008; Schwartzman-Morris and Putterman, 2012; Ngo et al., 2014). Consequently, a better understanding of sex differences may contribute to earlier diagnosis and better management of certain diseases (Brower, 2002; Lu et al., 2010). There is a growing body of evidence that has linked sex hormones to IBD susceptibility, disease severity, and disease progression (Khalili et al., 2013; Khalili et al., 2016). Studies have recognized differences in disease onset, subtypes, and progression patterns between females and males, suggesting the impact of sex on the occurrence and development of CD (Mazor et al., 2011; Severs et al., 2018; Shah et al., 2018; Shah et al., 2019). Although some studies have shown that sex hormones may affect the occurrence and development of diseases through farnesoid X receptor (FXR) (Rabinowitz et al., 2021), endoplasmic reticulum (ER) stress (van der Giessen et al., 2019) and intestinal flora (Son et al., 2019), the underlying mechanisms remain unclear. Sex disparities in the long-term prognoses of CD patients, including disease behaviour progression and the need for CD-related surgery, have not been studied using real-world, detailed cohort data, particularly in China. Therefore, based on a large retrospective cohort consisting of 611 patients, we attempted to recognize the risk factor profile for progression behaviour and surgery in female and male patients to better distinguish high-risk subgroups and optimize management along with the individualized treatment of CD patients.

## 2 Material and methods

### 2.1 Ethics declarations

The study protocol was abided by the principles of the Declaration of Helsinki and approved by the Institutional Review Board of Peking Union Medical College Hospital (PUMCH) (Ethical review number: S-K1100).

### 2.2 Patients

**FIGURE 1:**
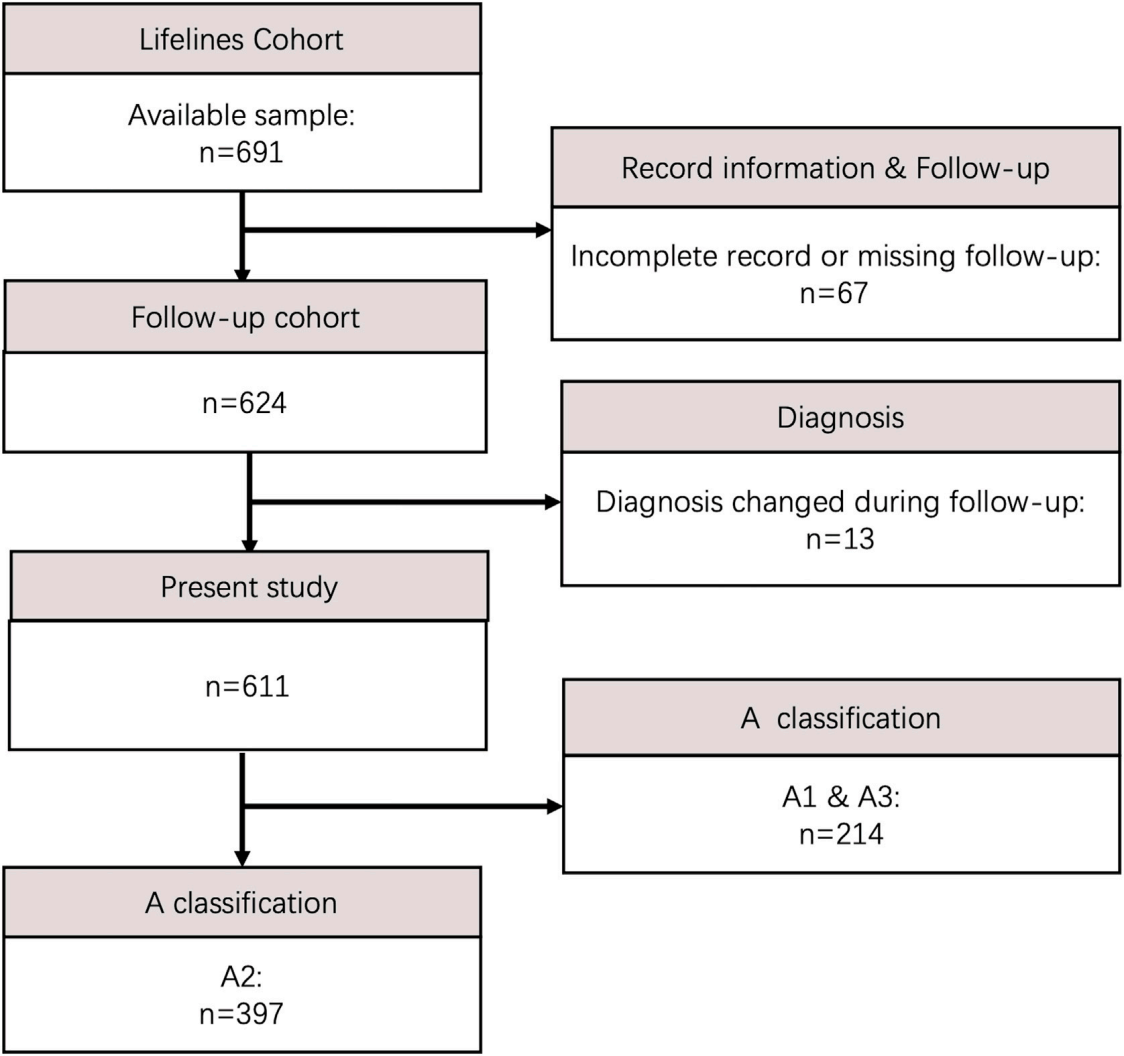
Flowchart of lifelines participant inclusion.

All inpatients admitted between January 2000 and December 2020 with a diagnosis of CD at the Peking Union Medical College Hospital (PUMCH) with detailed follow-up data were successively included in our analysis. Patients who satisfied the diagnostic criteria of the Chinese consensus (Inflammatory Bowel Disease GroupChinese Society of GastroenterologyChinese Medical Association, 2021) based on clinical, endoscopic, imaging, and pathological findings were enrolled. Patients with a malignancy history; autoimmune disease; or biological, immunomodulatory, or corticosteroid use before diagnosis were excluded. Those for whom the primary diagnosis was changed from CD to another disease or who were lost to follow-up were excluded. Patients lost to follow-up was identified when they refused to participate or could not be contacted with 1 year after diagnosis. Baseline information, including demographics, personal habits, medical history, clinical characteristics, onset time, complications, and treatments, was collected. The study enrolment flowchart is shown in Figure 1.

### 2.3 Assessment of outcomes

The clinical classification was based on the Montreal classification, and the Harvey Bradshaw index (HBI) was used to describe clinical disease activity (Best, 2006). Age classification included A1 (age at diagnosis <16), A2 (16 < age at diagnosis ≤40 years), and A3 (age at diagnosis>40) subtype. Disease behaviour progression was defined as the transition from an initial classification of B1 (nonstrictured/nonpenetrating) to B2 (strictured) or B3 (penetrating) (Bai et al., 2021). Location transition (Bai et al., 2021) was defined as the lesion location changing from L1 (terminal ileum)/L2 (colon) to L3 (ileo-colon)/L4 (upper gastrointestinal tract) or from L3 to L4. Multisystemic extraintestinal manifestations (EIMs) were defined as CD-related extraintestinal manifestations with the involvement of no less than two organs. CD-related surgery was limited to total or partial gastrointestinal resection or enterostomy due to complications or exacerbation of CD. Intestinal perforation was defined as a CD-related gastrointestinal penetrating lesion, including intestinal fistula but excluding anal fistula, which was confirmed by imaging, endoscopy, or surgery after a diagnosis of CD. Massive gastrointestinal bleeding (GIB) was defined as an acute bleeding event leading to a decline in the haemoglobin level by at least 2 mg/dl or a situation requiring blood transfusion support.

### 2.4 Follow-up protocol and definitions

Patients were followed up by a team responsible for information update. Electronic database was used for medical record review or telephone record every 2–3 years, and relevant information including medications, complications, progression behaviour or location classification, surgery, malignant tumour (including cancer in the intestinal tract or other organs) and death, and the times of occurrence were recorded. All patients were followed for at least 1 year following diagnosis, and the final follow-up report was completed in December 2021.

Primary outcomes were EIM(s), progression behaviour, and CD-related surgery after diagnosis. EIM-free survival (EFS) was defined as the period from diagnosis to the first EIM(s). Perforation-free survival (PFS) was defined as the time from diagnosis to the first intestinal perforation. Surgery-free survival (SFS) was defined as the period from diagnosis to abdominal surgery, including total or local intestinal resection.

### 2.5 Statistical analysis

All statistical analyses and displays were performed using SPSS software (version: 26.0, IBM, Chicago, IL, United States) and Microsoft ware power point. Categorical variables are expressed as frequencies and proportions, while continuous variables are expressed as medians (interquartile ranges, IQRs). Correspondingly, the groups were compared using Fisher’s exact test or the chi-square test. Kaplan–Meier survival curves and log-rank tests were used to compare EFS, PFS, and SFS. Univariate and multivariate Cox regression analyses were performed to estimate the hazard ratios (HRs) of the covariates that were potentially associated with the endpoints for each potential risk factor. As this study aimed to investigate disease progression and prognosis following diagnosis, in the Cox regression analyses, only the endpoints after the date of diagnosis were included. A two-sided *p* value of <0.05 was considered statistically significant.

## 3 Results

### 3.1 Patient demographics

Initially, 691 patients were diagnosed with CD in the study period. Of them, 13(1.8%) patients experienced a diagnostic change, and 67(9.7%) patients were lost to follow-up. Finally, a total of 611 patients who had ≥1 year of follow-up were enrolled in the present study (shown in Figure 1). There were 191(31.3%) female and 420(68.7%) male patients in this cohort, with a median follow-up time of 4.7(IQR 2.5–8.0) years. Among the 611 patients, 397 patients were diagnosed with the A2 classification (65.0%), followed by A3 (27.7%) and A1 (7.4%), with a median diagnosis age of 29.7 (IQR 21.4–42.2) years. One hundred and fifty (24.5%) patients experienced location transition and 174(28.5%) patients underwent disease behaviour progression during follow-up. In addition, 291(47.6%) patients had an EIM involving at least one organ during the disease course. Overall, 196(32.1%) participants experienced intestinal perforation after diagnosis. In total, 286(46.8%) patients underwent at least one CD-related surgery following its onset, including 140(22.9%) prediagnostic and 146(23.9%) postdiagnostic protocols. During follow-up, a total of 16(2.6%) patients developed malignant disease, and 21(3.4%) patients died. The clinical characteristics are shown in Table 1 and Supplementary Figure S1 in detail.

**TABLE 1:** Demographic and clinical characteristics of female and male CD patients at diagnosis and during follow-up.

Characteristics	All (*n* = 611)	Female (*n* = 191)	Male (*n* = 420)	*p*-value^e^
Follow-up time (year, median, IQR)	4.7 (2.5–8.0)	4.7 (2.4–8.8)	4.7 (2.5–7.8)	0.775
Time from symptom onset to diagnosis (months, median, IQR)	19.3 (6.6–54.8)	20.5 (6.7–61.2)	18.4 (6.6,52.0)	0.683
Smoked at diagnosis				
Current	69 (11.3)	3 (1.6)	66 (15.7)	0.000***
Former	73 (11.9)	1 (0.5)	72 (17.1)	
Never	469 (76.8)	187 (97.9)	282 (67.1)	
Age at diagnosis (years, median, IQR)	29.7 (21.4–42.2)	36.0 (24.2–48.6)	28.0 (20.7–39.0)	0.000***
Comorbidities (%)				
Diabetes mellitus	18 (2.9)	8 (4.2)	10 (2.4)	0.221
Hypertension	32 (5.2)	15 (7.9)	17 (4.0)	0.049*
Coronary heart disease	15 (2.5)	9 (4.7)	6 (1.4)	0.015*
Acute myocardial infarction	3 (0.5)	1 (0.5)	2 (0.5)	0.676^a^
Acute or chronic heart failure	4 (0.7)	4 (2.1)	0 (0.0)	0.097^a^
Urolithiasis	35 (5.7)	11 (5.8)	24 (5.7)	0.982
Appendectomy	90 (14.7)	33 (17.3)	57 (13.6)	0.231
Disease activity at diagnosis (%)				
Remission	85 (13.9)	27 (14.1)	58 (13.8)	0.993
Mild	135 (22.1)	41 (21.5)	94 (22.4)	
Moderate	288 (47.1)	90 (47.1)	198 (47.1)	
Severe	103 (16.9)	33 (17.3)	70 (16.7)	
A classification at diagnosis (%)				
A1	45 (7.4)	4 (2.1)	41 (9.8)	0.000***
A2	397 (65.0)	110 (57.6)	287 (68.3)	
A3	169 (27.7)	77 (40.3)	92 (21.9)	
L classification at diagnosis (%)				
L1	173 (28.3)	47 (24.6)	126 (30.0)	0.203
L2	148 (24.2)	52 (27.2)	96 (22.9)	
L3	252 (41.2)	76 (39.8)	176 (41.9)	
L4	38 (6.2)	16 (8.4)	22 (5.2)	
B classification at diagnosis (%)				
B1	278 (45.5)	86 (45.0)	192 (45.7)	0.431
B2	215 (35.2)	73 (38.2)	142 (33.8)	
B3	118 (19.3)	32 (16.8)	86 (20.5)	
Perianal involvement at diagnosis (%)	187 (30.6)	39 (20.4)	148 (35.2)	0.000***
Location transition^b^before surgery (%)	150 (24.5)	53 (27.7)	97 (23.1)	0.221
Disease behavior progression^c^ before surgery (%)	174 (28.5)	57 (29.8)	117 (27.9)	0.622
Overall medications usage (%)				
5-ASA	563 (92.1)	178 (93.2)	385 (91.7)	0.515
Glucocorticoid	448 (73.3)	138 (72.3)	310 (73.8)	0.587
IM	345 (56.5)	102 (53.4)	243 (57.9)	0.303
Biologic therapy	169 (27.7)	47 (24.6)	122 (29.0)	0.255
Time from diagnosis to first biologic therapy (months, median, IQR)	15.9 (3.1–46.0)	10.2 (1.1–43.4)	18.4 (3.4–49.7)	0.192
Overall occurrence of severe complications (%)	354 (57.9)	109 (57.1)	245 (58.3)	0.769
Obstruction	200 (32.7)	61 (31.9)	139 (33.1)	0.777
Perforation	196 (32.1)	50 (26.2)	146 (34.8)	0.035*
Massive GIB	73 (11.9)	26 (13.6)	47 (11.2)	0.392
Overall extra-intestinal manifestation (%)	291 (47.6)	93 (48.7)	198 (47.1)	0.764
Multisystemic extraintestinal manifestations^d^	113 (16.5)	49 (25.7)	64 (15.2)	0.002**
Overall CD-related surgery (%)	286 (46.8)	86 (45.0)	199 (47.3)	
Before diagnosis	140 (22.9)	46 (24.1)	93 (22.1)	0.697
After diagnosis	146 (23.9)	40 (20.9)	106 (25.2)	0.248
Overall malignancies (%)	16 (2.6)	10 (5.2)	6 (1.4)	0.006**
Total deaths (%)	21 (3.4)	6 (3.1)	15 (3.6)	0.787
Time from diagnosis to death (month, median, IQR)	48.2 (17.9–98.6)	46.4 (1.4–152.2)	48.6 (23.5–94.8)	0.85

*p < 0.05, **p < 0.01, ***p < 0.01.

a Fisher exact test.

b Location transition: Lesion location initially changed from L1 (terminal ileum)/L2 (colon) to L3 (ileo-colon)/L4 (upper gastrointestinal tract) or from L3 to L4.

c Disease behaviour progression: Behaviour classification changed from an initial classification of B1 (nonstrictured/nonpenetrating) to B2 (strictured) or B3 (penetrating) (Bai et al., 2021).

d Multisystemic extraintestinal manifestations: CD-related extraintestinal manifestations with the involvement of no less than two organs. IQR, interquartile range; IM, immunosuppressant GIB; 5-ASA, 5-aminosalicylic acid; PSC, primary sclerosing cholangitis.

e Comparision between female and male groups.

### 3.2 Sex disparities in clinical characteristics

#### 3.2.1 Baseline characteristics

The baseline characteristics of the females and males are summarized in Table 1. At diagnosis, male patients were significantly younger than female patients [28.0 (IQR: 20.2–39.0) vs. 36.0 (IQR: 24.2–48.6), *p* < 0.001] and were more likely to be exposed to cigarettes (32.8% vs. 2.1%, *p* < 0.001). In addition, a higher proportion of female patients reported a history of hypertension (7.9% vs. 4.0%, *p* < 0.05) and coronary heart disease (4.7% vs. 1.4%, *p* = 0.015). Regarding the Montreal classification, the A3 classification was more common in female patients (40.3% vs. 21.9%, *p* < 0.001), while perianal involvement occurred more often in male patients (20.4% vs. 35.2%, *p* < 0.001); no difference was observed in the location and behaviour classification between female and male patients at diagnosis. In accordance with the disparities in clinical manifestations, intestinal perforation occurred more frequently in males than in females (34.8% vs. 26.2%, *p* = 0.035). Moreover, female patients with an EIM were more likely to have multisystemic involvement (25.7% vs. 15.2%, *p* = 0.002). When prognosis was analysed, the incidence rate of malignancy was higher in female patients than in male patients (5.2% vs. 1.4%, *p* = 0.006).

#### 3.2.2 EIM(s)

**FIGURE 2:**
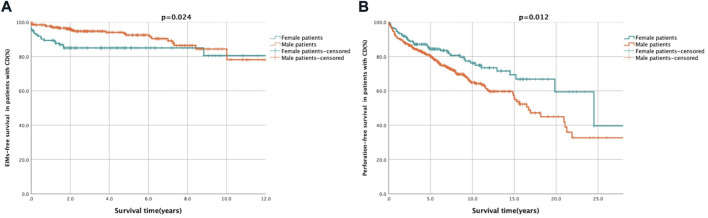
Kaplan-Meier survival curve presenting the EIMs- and perforation-free survival of female and male patients. Description: **(A)** EIMs-free after diagnosis survival of female and male patients. Female patients showed a trend towards shorter EIMs-free survival. **(B)** Perforation-free after diagnosis survival of female and male patients. Male patients showed a trend towards shorter perforation-free survival. EIMs-free survival was defined as the period from diagnosis to occurrence of EIMs. Perforation-free survival was defined as the period from diagnosis to occurrence of CD related gastrointestinal penetrating lesion.

Two hundred and ninety-one patients with EIMs were selected for a substudy, including 93 female patients and 198 male patients. Of them, there were 113 patients (49 females and 64 males) experienced multisystemic EIMs (involvement of no less than two organs), with a significantly higher proportion in females (25.7% vs. 15.3%, *p* = 0.002, Table 1). According to the univariate and multivariate analyses (Table 2), male patients [HR: 0.52, 95% confidence interval (CI): 0.29–0.93, *p* < 0.05] had a lower probability of developing EIMs with multiple organ involvement than female patients. In addition, male sex was the only independent protective factor against multisystemic EIMs (HR: 0.52, 95% CI: 0.29–0.94, *p* = 0.03). The Kaplan–Meier survival analysis indicated that male sex was associated with longer EFS (HR: 0.52, *p* = 0.024, Figure 2A).

**TABLE 2:** Univariate and multivariate Cox regression analysis of risk factors for multisystemic EIMs and intestinal perforation in CD patients.

Variables	Univariate analysis				Multivariate analysis			
	Multisystemic EIMs^a^ (*n* = 113)		Perforation (*n* = 196)		Multisystemic EIMs (*n* = 113)		Perforation (*n* = 196)	
	HR (95% CI)	*p*-value	HR (95% CI)	*p*-value	HR (95% CI)	*p*-value	HR (95% CI)	*p*-value
Sex (male)	0.52 (0.29–0.93)	0.03*	1.71 (1.13–2.60)	0.01*	0.52 (0.29–0.94)	0.03*	1.85 (1.20–2.84)	0.01*
Smoked at diagnosis								
Never	Ref	—	Ref	—	—	—	—	—
Former	0.88 (0.34–2.26)	0.80	0.77 (0.46–1.27)	0.31	—	—	—	—
Current	1.43 (0.46–4.48)	0.53	1.19 (0.61–2.32)	0.61	—	—	—	—
Duration (year)	1.00 (0.99–1.00)	0.57	1.00 (0.99–1.00)	0.72	1.00 (0.99–1.00)	0.59	—	—
Disease activity at diagnosis								
Severe	Ref	—	Ref	—	—	—	Ref	—
Moderate	0.80 (0.33–1.95)	0.63	0.32 (0.16–0.63)	0.00**	—	—	0.33 (0.17–0.66)	0.00**
Mild	0.38 (0.14–1.01)	0.05	0.44 (0.26–0.74)	0.00**	—	—	0.45 (0.27–0.78)	0.00**
Remission	0.59 (0.29–1.19)	0.14	0.55 (0.36–0.83)	0.01*	—	—	0.56 (0.36–0.86)	0.01*
A classification at diagnosis								
A1	Ref	—	Ref	—	—	—	Ref	—
A2	1.02 (0.40–2.60)	0.97	0.52 (0.22–1.25)	0.14	—	—	0.34 (0.14–0.82)	0.02*
A3	0.50 (0.27–0.94)	0.03*	1.03 (0.69–1.52)	0.90			0.82 (0.50–1.23)	0.35
L classification at diagnosis								
L1	Ref	—	Ref	—	—	—	—	—
L2	0.48 (0.16–1.51)	0.21	0.46 (0.19–1.07)	0.07	—	—	—	—
L3	0.70 (0.23–2.14)	0.54	1.01 (0.45–2.28)	0.97	—	—	—	—
L4	0.47 (0.16–1.43)	0.18	1.14 (0.52–2.50)	0.75	—	—	—	—
Perianal lesion	0.93 (0.49–1.77)	0.83	1.53 (1.08–2.19)	0.02*	—	—	1.23 (0.84–1.81)	0.29
Transition of L^b^	1.48 (0.81–2.71)	0.20	1.54 (1.07–2.20)	0.02*	—	—	1.42 (0.98–2.06)	0.06
IM	1.22 (0.67–2.24)	0.52	1.07 (0.75–1.52)	0.72	1.00 (0.53–1.88)	0.99	—	—
Biologic therapy	0.56 (0.25–1.25)	0.16	1.20 (0.82–1.74)	0.35	0.55 (0.24–1.22)	0.14	—	—
Glucocorticoid	2.23 (0.95–5.28)	0.07	0.85 (0.58–1.27)	0.43	2.29 (0.92–5.69)	0.07	—	—

*p < 0.05, **p < 0.01, ***p < 0.01.

Ref, reference category. Medication usage was defined as therapy used before behavior progression.

a CD-related extraintestinal manifestations with no less than two organs involvement.

b Transition of location classification before behavior progression. IM, immunosuppressant; EIMs, extra-intestinal manifestations.

#### 3.2.3 Disease progression

The impact of sex on intestinal perforation (Table 2) in a substudy involving 196 patients (50 females and 146 males) was also analysed. The Cox regression analysis demonstrated that male sex was associated with an increased likelihood of a penetrating combination (HR: 1.71, 95% CI: 1.13–2.60, *p* = 0.01) and was an independent risk factor for gastrointestinal perforation following diagnosis (HR: 1.85, 95% CI: 1.20–2.84, *p* = 0.01). The Kaplan–Meier curve revealed that male patients were more likely to have shorter PFS after diagnosis than female patients (HR: 1.71, *p* = 0.012, Figure 2B).

### 3.3 Subgroup analysis of surgery-related factors in patients with an A2 classification

**FIGURE 3:**
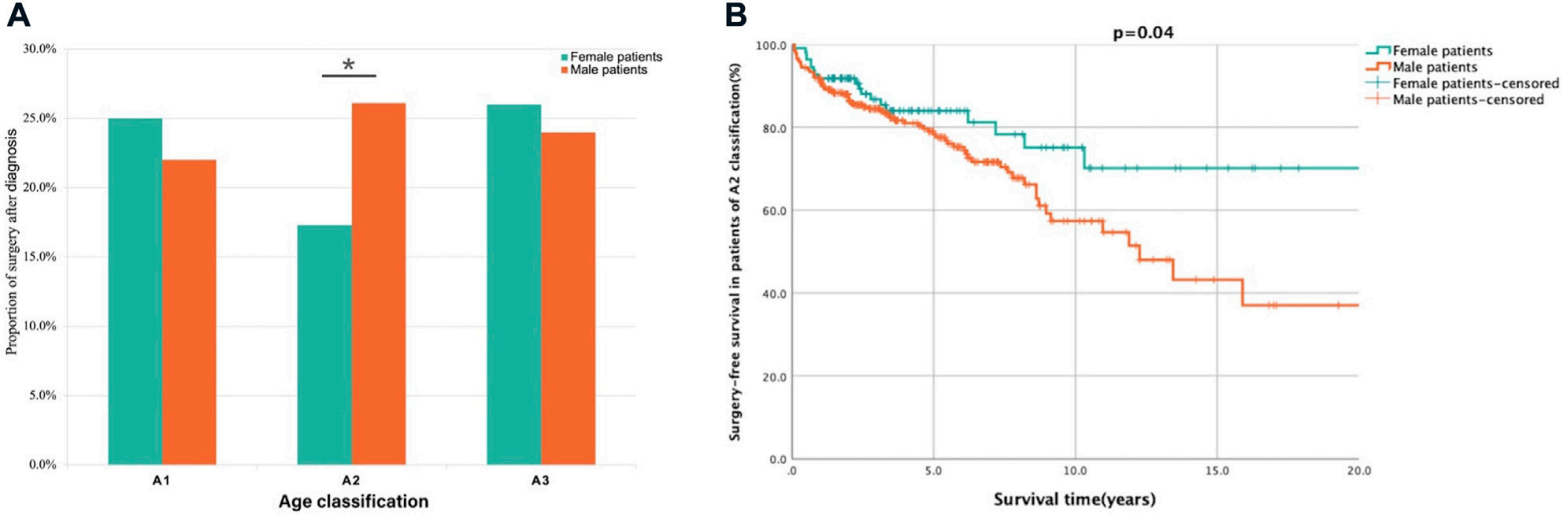
Analysis of surgery proportion and survival in female and male patients based on A classification. Description: **(A)** The proportion of surgery after diagnosis in male patients was lower than female patients in A1 and A3 classification but significantly higher in A2 classification. **(B)** The surgery-free survival was shorted in male patients of A2 classification. *p < 0.05. Surgery-free after diagnosis survival was defined as the period from diagnosis to CD related surgery.

As shown in Table 1 patients were identified as A2 subtype (110 females and 287 males) at diagnosis, with 94(23.7%) patients undergoing surgery following diagnosis. From the analysis of clinical characteristics of patients (Table 1), the patients in A2 subtype (65.0%) were the majority in our centre, and the results suggested significant sex disparities in the Montreal age classification of CD. To further analyse the correlation between sex and long-term outcomes, univariate and multivariate analyses were performed (Table 3). As shown in Figure 3A, though the female patients had higher proportions of surgery following diagnosis both in A1 (22.0% vs. 25.0%) and A3 (24.0% vs. 26.0%) subtype, there were no significant differences, respectively. However, there was a significantly increased likelihood of surgery in males relative to female patients in A2 subtype (26.1% vs. 17.3%, *p* = 0.04). Multivariate Cox regression analysis revealed that male sex (HR: 1.80, 95% CI: 1.07–3.01, *p* = 0.028) was an independent risk factor for surgery in the patients of A2 subtype. Notably, male patients of A2 subtype had shorter SFS than female patients (HR: 1.69, *p* = 0.04, Figure 3B).

**TABLE 3:** Univariate and multivariate Cox regression analysis of risk factors for surgery after diagnosis among CD patients based on A classification.

Variables	Univariate analysis						Multivariate analysis	
	A1 (*n* = 10)		A2 (*n* = 94)		A3 (*n* = 42)		A2 (*n* = 94)	
	HR (95% CI)	*p*-value	HR (95% CI)	*p*-value	HR (95% CI)	*p*-value	HR (95% CI)	*p*-value
Sex (male)	0.42 (0.05–3.5)	0.42	1.69 (1.02–2.8)	0.042*	0.98 (0.53–1.79)	0.94	1.80 (1.07–3.01)	0.028*
Smoked at diagnosis								
Never	Ref	—	Ref	—	Ref	—	—	—
Former	0.17 (0.02–1.48)	0.108	0.91 (0.47–1.78)	0.791	1.31 (0.51–3.4)	0.579	—	—
Current	1.37 (0.08–23.33)	0.827	1.76 (0.8–3.91)	0.163	2.34 (0.78–7.06)	0.13	—	—
Disease activity at diagnosis								
Remission	Ref	—	Ref	—	Ref	—	Ref	—
Mild	1.90 (0.15–24.18)	0.62	0.38 (0.17–0.89)	0.026	0.39 (0.15–1.04)	0.059	0.40 (0.17–0.96)	0.037*
Moderate	1.19 (0.19–7.37)	0.854	0.42 (0.22–0.82)	0.012*	0.2 (0.07–0.63)	0.006**	0.36 (0.18–0.73)	0.004**
Severe	1.63 (0.28–9.55)	0.586	0.75 (0.47–1.22)	0.252	0.4 (0.19–0.82)	0.012*	0.71 (0.43–1.17)	0.171
L classification at diagnosis								
L1	Ref	—	Ref	—	Ref	—	—	—
L2	0.56 (0.14–2.13)	0.961	0.69 (0.24–2.02)	0.50	0.73 (0.21–2.59)	0.629	—	—
L3	0.92 (0.25–2.89)	0.96	1.07 (0.37–3.06)	0.905	0.97 (0.28–3.41)	0.959	—	—
L4	1.05 (0.2–3.68)	0.967	1.27 (0.45–3.53)	0.653	0.72 (0.21–2.54)	0.612	—	—
B classification at diagnosis								
B1	Ref	—	Ref	—	Ref	—	—	—
B2	0.18 (0.04–0.92)	0.039*	0.52 (0.3–0.88)	0.015*	0.46 (0.19–1.08)	0.073	—	—
B3	0.5 (0.11–2.39)	0.388	0.95 (0.56–1.62)	0.84	0.82 (0.38–1.74)	0.601	—	—
Perianal lesion	0.69 (0.19–2.47)	0.571	1.1 (0.73–1.67)	0.647	0.59 (0.23–1.5)	0.266	0.82 (0.51–1.32)	0.352
Transition of L^b^	2.4 (0.69–8.29)	0.168	1.29 (0.84–1.97)	0.249	2.18 (1.18–4.02)	0.013*	—	—
Behavior progression^a^	3.28 (0.9–12.01)	0.073	1.86 (1.23–2.79)	0.003**	2.08 (1.13–3.81)	0.019*	1.94 (1.27–2.97)	0.002**
Surgery before diagnosis	2.12 (0.25–17.67)	0.488	0.82 (0.49–1.37)	0.445	0.43 (0.1–0.9)	0.025*	0.73 (0.43–1.26)	0.264
IM	0.91 (0.18–4.66)	0.913	1.80 (1.15–2.82)	0.01*	1.26 (0.68,2.34)	0.466	2.02 (1.25–3.27)	0.004**
Biologic therapy	2.31 (0.61–8.72)	0.219	0.99 (0.65–1.54)	0.991	1.31 (0.58–2.95)	0.521	—	—
Glucocorticoid	0.89 (0.19–4.29)	0.886	0.72 (0.46–1.13)	0.156	0.85 (0.44–1.64)	0.627	0.51 (0.31–0.84)	0.008**

*p < 0.05, **p < 0.01, ***p < 0.01.

Ref, reference category. Medication usage was defined as therapy used before surgery after diagnosis.

a Behavior progression before surgery after diagnosis. IM, immunosuppressant.

b Transition of location classification before surgery after diagnosis.

## 4 Discussion

Despite the acknowledged sex disparities in the clinical presentations of CD (Gupta et al., 2007; Coughlan et al., 2017; Severs et al., 2018), little is known about the impact of sex on the outcomes of CD. Here, based on our retrospective CD cohort of 611 patients, we compared the clinical characteristics and prognoses between female and male patients with CD.

Female and male CD patients had different baseline demographic and clinical features. Male patients were diagnosed at a younger age than females in our study, and the male to female ratio was 2.20:1, consistent with other studies on sex differences in the CD prevalence and incidence in Asian populations (Yang et al., 2001; Leong et al., 2004; Prideaux et al., 2012; Severs et al., 2018; Shah et al., 2018). Our data showed that more male patients were diagnosed with the A1 and A2 classification than female patients, while the proportion of the A3 classification was higher in females than in males. The sex difference in age classification seemed to coincide with age-related changes in sex hormones levels, revealing a potential correlation between sex hormones levels and CD onset. We found that perianal involvement was more prevalent in men, which was in line with previous findings that males have a greater probability of a complex course of CD than females (Mazor et al., 2011; Severs et al., 2018; Heath et al., 2021). Previous studies have shown that females with CD are at high risk of upper gastrointestinal involvement (Greuter et al., 2018). Some studies also report a significant association between male sex and ileal lesions in CD patients (28% in males and 20% in females) (Severs et al., 2018). Nevertheless, we did not detect any significant differences in lesion location in this study.

EIMs contribute greatly to the disease burden of CD; EIMs are known to exhibit a clear sex-based distribution and a partial relationship with disease activity. Overall, EIMs are more common in female patients with IBD (Wagtmans et al., 2001; Halling et al., 2017; Severs et al., 2018). Peripheral joint lesions and cutaneous manifestations, including gangrenous pyoderma, erythema nodosum, and ocular diseases, occur more frequently in females, whereas ankylosing spondylitis and primary sclerosing cholangitis occur more frequently in males (Bernstein et al., 2001). Although no sex disparities in the organ distribution of EIMs were observed in this work, we found a greater probability of a concurrent involvement of multiple systems in females than in males among the patients with EIMs. Interestingly, male sex was a protective factor against multisystemic EIMs, and female patients appeared to develop EIMs earlier than males, suggesting a potential sex disparity in the clinical features and disease development.

In previous studies, male CD patients are prescribed prednisone more often (Severs et al., 2018), while budesonide is more often prescribed in females (Lie et al., 2017). Additionally, females received biological therapy later and used antitumor necrosis factor-α drugs less frequently, due to more side effects leading to lower adherence, than males (Greuter et al., 2020). Whereas men required a greater degree of dose escalation than females (Billioud et al., 2011). There were no significant differences in the drug regimens between male and female patients in our data, although females received biological therapy earlier than males. Studies have shown that male sex, upper gastrointestinal involvement, and steroid use are associated with the early transition from classification B1 to B2 or B3 (31). Although no sex disparities in disease behaviour translations were found in this study, unlike other studies that reported steroid use as a risk factor for surgery (Peyrin-Biroulet et al., 2012), exposure to steroids significantly reduced the risk of surgery in CD patients with A2 classification.

The results of two large prospective studies by the Dutch IBD Biobank showed enhanced rates of ileal and small bowel resection in male CD patients (Severs et al., 2018), and a study by the Mayo Clinic also reported male sex as an independent predictor of major abdominal surgery (Peyrin-Biroulet et al., 2012). However, a separate study (Wagtmans et al., 2001) showed that female patients underwent ileal resection more often than male patients, with early postoperative recurrence. Although a significant impact of sex on CD-related surgery was not found, our data confirmed that intestinal perforation, one of the most serious complications of CD, occurred more frequently in male patients, leading to shorter perforation-free survival (PFS). Male sex was demonstrated to be an independent risk factor for penetrating lesions, suggesting a relationship between sex and disease progression. Interestingly, among patients diagnosed with the A2 classification, male sex was found to be an independent significant risk factor for abdominal surgery, and male patients had shorter SFS than females, implying a worse prognosis.

Indeed, sex disparities in disease outcomes among CD patients may be related to hormonal, immune, genetic, epigenetic, or behavioural factors (e.g., diet and smoking behaviour). Males are at risk of more severe disease activity (Magro et al., 2014; Hausmann and Blumenstein, 2015), owing to differences and the intersection of sex hormones with immune system activation and gut physiology. Additionally, sex-based environmental differences also account for the disparities, including diet and smoking behaviour (Ngo et al., 2014). Differences in health-seeking behaviour can lead to differences in treatment and the availability of surgical intervention options. Consequently, delayed access to medical care, a behaviour more common among men, may have a great effect on severe CD development. Conversely, lower rates of surgery in females may be related to their concerns about the risks of surgery, postoperative body image, or the impact of surgery on fertility (Muller et al., 2010; Sceats et al., 2019).

The pathophysiology underlying sex differences in the epidemiology and disease progression of CD remains largely unknown. Sex hormones based on sex-related genes are supposedly a potentially decisive factor. Epidemiological studies have shown a higher rate of CD occurrence in families of female patients (particularly involving a genetic pattern of transmission by females) and a greater IBD incidence rate in patients with the XO genotype (Turner’s syndrome) (Arulanantham et al., 1980), suggesting that X-chromosome-linked antecedent events may contribute to sex-specific differences in CD incidence. In addition, the use of exogenous hormones, including oral contraceptives, is substantially associated with the progression and risk of CD (Cornish et al., 2008; Khalili et al., 2013; Khalili et al., 2016). Previous studies have suggested a correlation between sex hormones and the incidence and progression of CD. As acknowledged, patients diagnosed with the A2 classification were in a phase during which an important shift in the maturation of sex hormones occurred simultaneously (Jašarević et al., 2016). In our study, sex disparities in the probability of surgery and SFS were observed in those with the A2 classification but not in those with the A1 or A3 classification, indicating a worse prognosis at a similar age, suggesting a relationship between sex hormones and disease development. Considering the sex difference in the proportion of those with an A classification, sex hormones likely have an impact not only on CD onset but also on disease progression. A study showed that hyperglycaemia exacerbated the pathological conditions in male animal models, while the female control group had a milder disease course (Singla et al., 2021), supporting the potentially important role of sex hormones in disease development.

Some approaches by which sex hormones affect the occurrence and development of CD have been proposed. A recent study (Rabinowitz et al., 2021) reported the effect of sex hormones on genetic variations in the farnesoid X receptor (FXR). After correcting for confounding factors in the retrospective cohort, female CD patients with the FXR-1G >T genotype were at higher risk of early surgery; oestrogen may contribute to the inhibition of FXR-1 activity through oestrogen receptor signalling, suggesting that oestrogen may mitigate the risk of surgery through this suppression. Our findings demonstrated that male sex in the A2 classification was associated with a higher frequency of surgery and shorter SFS, in accordance with the abovementioned study. However, whether this mechanism contributes to the pathogenesis of surgical risk in CD needs to be further validated. Furthermore, sex hormones have been found to have beneficial effects on disease activity by positively modulating intestinal epithelial barrier function by reducing endoplasmic reticulum (ER) stress and proinflammatory cytokine production in IBD (van der Giessen et al., 2019). Moreover, a previous study reported that sex hormones directly modulated the metabolism of bacteria through steroid receptors, including oestrogen receptor beta (Menon et al., 2013). Sex differences in the diversity and species of intestinal flora associated with the development of IBD were observed (Son et al., 2019). Because of the important role of the gut microbiota in IBD development (Lee and Chang, 2021) and the correlation between sex hormones and the gut microbiota, a potential effect of sex hormones on disease progression through modulation of the microbiota is worth further exploration.

To our acknowledgement, the present study was a large, local, single-centre, retrospective cohort study that assessed the long-term prognosis of CD patients in China using real-world data. Remarkably, it is the first study focusing on the impact of sex disparity on disease progression and surgery-related risk factors in patients with CD. Acknowledgement of the effects of sex on the factors associated with CD prognosis is an important step towards the early identification of groups at high risk for poor outcomes. This study could provide future insights into appropriate management and individualized treatment regimens for patients with CD. The sex differences in the disease progression and prognosis of CD also suggested that the mechanism of CD occurrence and development may be associated with sex hormone-related pathways, and further laboratory studies are needed to confirm our findings. However, our study has some limitations. Firstly, this retrospective study in single centre lacked consistency in data collection as well as control data for making comparisons. Hence, the findings should be interpreted with caution. Secondly, the samples in this study were also limited to hospitalized patients with CD as primary diagnosis which may lead to some bias. The last, as CD is a disease with long course, the 5 years follow up period may not be sufficiently long enough to adequately capture and delineate the disease trajectory. Therefore, the findings require further validation in a long-term multicentre prospective cohort study.

## 5 Conclusion

Our findings have revealed differences in clinical characteristics, disease progression behaviour, and outcomes between female and male patients with CD. Female sex was significantly independently associated with earlier multisystemic EIMs and later intestinal perforation. Male sex was independently related to a poor prognosis among those with the A2 classification. These findings suggest that we need to pay more attention to sex disparities in CD in clinical practice to identify high-risk patients. Moreover, early interventions for the subgroups with high-risks of disease progression and CD-related surgery may improve the long-term prognosis. To validate our findings, future prospective, national cohort studies are needed. Further laboratory studies on the sex hormone pathway will help to further reveal the pathogenesis and progression of CD.

## Data Availability

The original contributions presented in the study are included in the article/Supplementary Material, further inquiries can be directed to the corresponding author.

## Supplementary material

SUPPLEMENTARY FIGURE S1 Comparison of partial clinical characteristics between female and male patients. Significant *p*-value <0.05. **p* < 0.05, ***p* < 0.01, ****p* < 0.001. EIMs, extra-intestinal manifestations.
