# Supplementary material for: Sex-specific comparison of clinical characteristics and prognosis in Crohn’s disease: A retrospective cohort study of 611 patients in China
Source: Front Physiol. 2022 Sep 29;13:972038. doi: 10.3389/fphys.2022.972038 (PMC9557081; doi:10.3389/fphys.2022.972038)
Supplement: Supplementary file 1 [file Table1.DOCX]

Figure legend:

Supplementary Figure 1. Comparison of partial clinical characteristics between female and male patients

Significant *p*-value <0.05. ^*^p＜0.05,^**^p＜0.01,^***^p＜0.001. EIMs=Extra-intestinal manifestations.
