# Supplementary figures and images for: Sex-specific comparison of clinical characteristics and prognosis in Crohn’s disease: A retrospective cohort study of 611 patients in China
Source: Front Physiol. 2022 Sep 29;13:972038. doi: 10.3389/fphys.2022.972038 (PMC9557081; doi:10.3389/fphys.2022.972038)

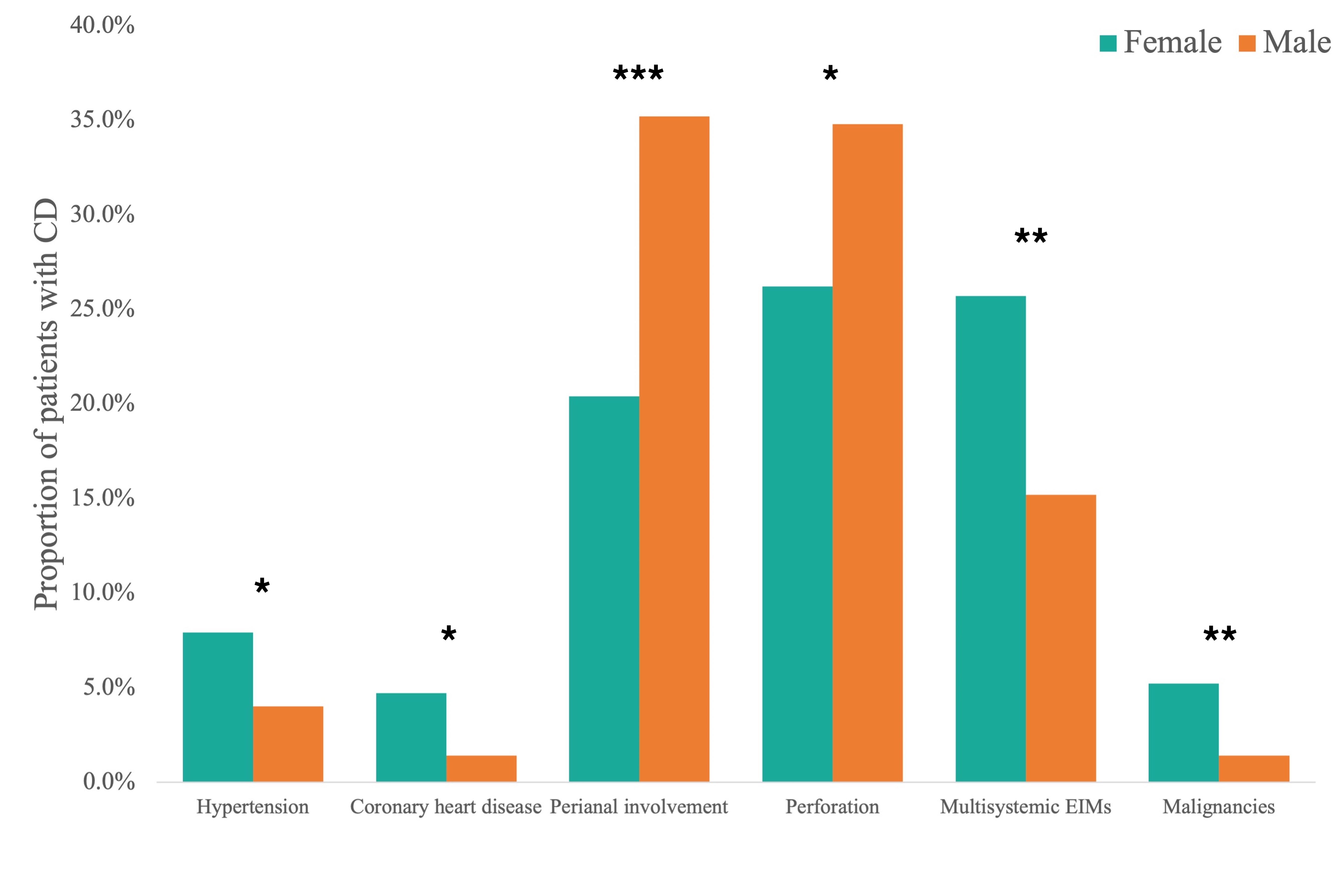

Supplement: Supplementary file 2 [file Image1.JPEG]
